# Cryptic Speciation and Chromosomal Repatterning in the South African Climbing Mice *Dendromus* (Rodentia, Nesomyidae)

**DOI:** 10.1371/journal.pone.0088799

**Published:** 2014-02-13

**Authors:** Emanuela Solano, Peter J. Taylor, Anita Rautenbach, Anne Ropiquet, Riccardo Castiglia

**Affiliations:** 1 Department of Biology and Biotechnologies “Charles Darwin”, Sapienza University of Rome, Rome, Italy; 2 Department of Ecology & Resource Management, School of Environmental Sciences, Thohoyandou, South Africa; 3 School of Life Sciences, University of KwaZulu-Natal, Durban, South Africa; 4 Imperial College London Silwood Park Campus, Ascot, Berks, United Kingdom; University of Florence, Italy

## Abstract

We evaluate the intra- and interspecific diversity in the four South African rodent species of the genus *Dendromus*. The molecular phylogenetic analysis on twenty-three individuals have been conducted on a combined dataset of nuclear and mitochondrial markers. Moreover, the extent and processes underlying chromosomal variation, have been investigated on three species by mean of G-, C-bands, NORs and Zoo-FISH analysis. The molecular analysis shows the presence of six monophyletic lineages corresponding to *D. mesomelas*, *D. mystacalis* and four lineages within *D.* cfr. *melanotis* with high divergence values (ranges: 10.6% – 18.3%) that raises the question of the possible presence of cryptic species. The first description of the karyotype for *D. mesomelas* and *D. mystacalis* and C- and G- banding for one lineage of *D.* cfr. *melanotis* are reported highlighting an extended karyotype reorganization in the genus. Furthermore, the G-banding and Zoo-FISH evidenced an autosome-sex chromosome translocation characterizing all the species and our timing estimates this mutation date back 7.4 mya (Late Miocene). Finally, the molecular clock suggests that cladogenesis took place since the end of Miocene to Plio-Pleistocene, probably due to ecological factors, isolation in refugia followed by differential adaptation to the mesic or dry habitat.

## Introduction

Rodentia are the most diversified mammals, with more than two thousand species [1]. This number is ever increasing, as new species are continually described through molecular phylogenetics and DNA barcoding studies, revealing frequently the presence of cryptic species [2]. Besides these molecular approaches, the study of the karyotype in rodents contributed in delimiting species [3] and the chromosomal rearrangements are suspected to have a role in the speciation itself [4]–[6].

This study focuses on the South African species of the climbing mice, genus *Dendromus* Smith, 1829 (Rodentia, Nesomyidae), that belong to the Dendromurinae, currently restricted to Africa. In this region, several rodents lineages have been molded by complex events during Miocene and Plio-Pleistocene, yet not completely understood [7]. While the phylogenetic positions of *Dendromus* and of Dendromurinae in the family has been defined [8]–[9], the evolutionary relationships among the constituent species are unresolved and the species limits require an extensive revision.

**Figure 1:**
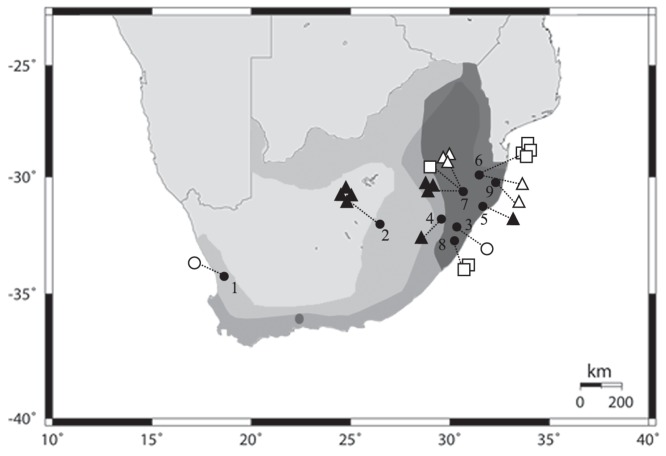
*Dendromus* species distribution in South Africa. The scale of grey areas (from light to dark tone) indicate *D. melanotis*, *D. mesomelas* and *D. mystacalis* respectively. The numbers indicate the sample localities listed in Table S1. The symbols represent the species sampled in each locality - Black triangles: *D. melanotis*; White circles: *D. mesomelas*; White squares: *D. mystacalis*; White triangles: *Dendromus sp*.

Musser and Carleton [10] recognized 12 species, but recently, an additional species was described from Guinea [11]. Four species, with partially overlapping ranges, occur in South Africa [12] (Fig. 1): *D. melanotis* Smith, 1829, *D. mesomelas* Brants, 1827, *D. mystacalis* Heuglin, 1863 and *D. nyikae* Wroughton, 1909.

The four species cannot be all easily distinguished morphologically since some diagnostic character may be equivocal. However, even if sympatric, the species show a certain degree of ecological separation. *Dendromus melanotis*, the grey climbing mouse, is a morphologically variable species with a wide distribution in South Africa and lives in drier areas with short grassland. *Dendromus mesomelas*, Brants's climbing mouse, overlaps with *D. melanotis* except for the northern part of the country where it is absent (Fig. 1). *D. mesomelas* is markedly different from the sympatric species in pelage colour and other morphological traits. *Dendromus mystacalis*, the chestnut climbing mouse, occurs predominantly in the eastern parts of South Africa, is smaller than *D. mesomelas* but very similar in morphology and they both prefer wet zones and long grass. Finally, *Dendromus nyikae*, the Nyika climbing mouse, is dark-furred and large-bodied. It has a distribution mostly outside of South Africa; it fringes into the Limpopo Province in the north-eastern part of South Africa. *D. nyikae* is considered closely related to *D. melanotis* and sometimes they can be morphologically confused since the differences mainly concern the size and sometimes the pelage colour pattern.

Cytogenetic data for *Dendromus* are sparse and are mainly limited to standard stained karyotypes [13]–[15]. Matthey [13], comparing diploid numbers, shape of chromosomes and fundamental number in related genera argued that complex rearrangements occurred in the speciation of genus. Furthermore, Dippenaar et al. [15] recorded a 2n = 52 (NFA = 62) for a *D. melanotis* from the Kalahari region of South Africa. Only recently, a C- and G-banded karyotype was studied in *D. lachaisei* from Guinea [11]. These data suggest that considerable chromosomal variability exists among the recognized species, with variation recorded from 2n = 30 to 2n = 52.

The aim of this investigation was to evaluate the intra- and interspecific genetic diversity in species of *Dendromus* and to estimate their phylogenetic relationships. We also provide the first comparative cytogenetics study for the genus including the first description of the karyotype for *D. mesomelas* and *D. mystacalis* and the first C- and G-banding patterns for a single lineage of *D.* cfr. *melanotis*. The extent and processes underlying temporal chromosomal variation was estimated by a molecular phylogenetic analysis.

## Materials and Methods

### Samples

Twenty-three *Dendromus* specimens were analysed from nine localities in South Africa (Table 1, Fig. S1). The specimens, identified using external traits and skull morphology (see [16]), were assigned to *D. melanotis* (N = 9), *D. mesomelas* (N = 2) and *D. mystacalis* (N = 7). However, for five specimens morphologically similar to *D. melanotis* (indicated herein as *Dendromus* ***s*** *p*.), the identification using existing taxonomic keys was equivocal.

**Table 1:** Pairwise genetic distances.

	L1	L2	L3	L4	C2	C3
L1	-	-	-	-	-	-
L2	0,183	-	0,034	0,041	0,061	0,064
L3	0,141	0,159	-	0,024	0,063	0,067
L4	0,166	0,171	0,106	-	0,068	0,072
C2	0,205	0,212	0,200	0,196	-	0,006
C3	0,193	0,190	0,170	0,163	0,105	-

The pairwise genetic divergence (net between-group mean distances) among the four lineages of the clade C1 (lineages L1-L4) and the clades C2 and C3 based on cyt *b* (lower triangle) and Fib I7 (upper triangle).

### Ethic statement

No protected species were used in this study. The three animals used for the cytogenetic procedures were live trapped with Sherman traps placed in transects on the ground. The bait was a mixture of peanut butter, apples and oatmeal. Traps were set in the late afternoon and inspected in the early morning. The sampling was conducted in accordance with regulations of collecting permits issued to the Durban Natural Science Museum, PJT and AR by Ezemvelo KZN Wildlife permits Office, conform to the Animal Protection Act (1962) and the National Environmental Management: Protected Areas Act 57 (2003). Animal handling procedures were approved by the animal ethics sub-committee of the University of Kwazulu-Natal. The capture and euthanasia of animals were performed in accordance with prescribed guidelines of the American Society of Mammalogists for the use of wild mammals in research, outlined in Sikes and Grannon (2011). Each animal was anesthetized and euthanized at the capture site by overdose of inhalant anesthetic according to the “Guideline to the care and use of animals in research and teaching” of the Animal Ethics Committee of the University of KwaZulu-Natal. All other samples were sourced from existing tissue collections associated with museum collection. Specimens were deposited in the Durban Natural Science Museum, South Africa. Voucher numbers and grid references to the collection localities are reported in Table S1.

### Sequencing of the molecular markers cyt b and Fgb I7

DNA was extracted (DNeasy Tissue Kit, Quiagen) from EtOH-preserved tissue of all specimens of *Dendromus* as well as of two specimens used as outgroups (see subsequent details on the outgroup choice), *Eliurus minor* and *Nesomys rufus* (Nesomyinae). A fragment of about 1100 bp, representative of almost the entire mitochondrial Cytochrome B (cyt *b*) gene, was amplified for 13 specimens and shorter fragments of cyt *b* (from 990 bp to 662 bp) were amplified for another eight specimens. For two specimens (code DM8518 and DM2596, material from old museum collections), it was possible to amplify only ∼300 bp of cyt *b* on account of the degraded state of the DNA. All PCR analyses were performed using the L14724, H15162, L15494 and H15915 universal mammal primers [17] in different combinations with the PCR amplification described therein. The corresponding primers were used in the sequencing reactions. The numbers of variable sites, parsimoniously informative sites, and the nucleotide composition [17] at all the three codon positions were calculated with DNASp v.5 [18]. Genetic distances (net between-group mean) between clades/lineages were estimated by the Kimura two-parameter model as implemented in MEGA v.5.0.5. [19].

The nuclear β-fibrinogen intron 7 (Fgb I7) was amplified for a subset of 14 *Dendromus* specimens and two Nesomyinae to assess the consistency of the phylogeny in maternal and biparentally hereditable markers. The Fgb I7 have been chosen for being a rapidly evolving intron and then suitable for the taxonomic level investigated herein and, at the same time, comparable with the cyt *b*. The PCR protocol cycles as described in Seddon et al. [20] were applied, and the same primers used for the amplification described herein (BFibR1 and BFibR2) were used for the sequencing. A fragment (about 650 bp) comprising most of intron7 was amplified and sequenced for all 16 specimens (Table S1) and for two of the outgroups, *E. minor* and *N. rufus*.

The sequences were checked in Sequencer v.5.0 and aligned using Se–Al v.0.1 [21]. In total, 23 sequences for cyt *b* and 16 sequences for Fgb I7 are deposited in GenBank (Accession nos KF811213 - KF811251, see Table S1).

### Phylogeny and molecular clock

The outgroups were chosen in order to use the same species for the two datasets and, at the same time, to have the calibration points for the molecular clock analysis (see later in this section). According to the literature, the sister taxon of *Dendromus* is *Steatomys* and the Nesomyinae are the outgroup to the Dendromurinae [9], [22]. For the cyt *b* dataset, sequences of *Steatomys parvus*, *Steatomys sp.* and the Nesomyinae *Eliurus minor* and *Nesomys rufus* were available in GenBank (Accession nos. AF160599, AJ010562, HM2230720 and AF160592). Since only one paleontological calibration point was available (*Dendromus* - *Steatomys*), we added sequences of the Muroidea *Apodemus sylvaticus, Mus domesticus* and *Rattus norvegicus* (Accession nos. AJ631968, AB125773 and EU273707) to the cyt *b* analysis to provide further splitting times. For the Fgb I7 dataset, we used the same outgroup specimens (with the exception of *Steatomys*, since the Fgb I7 sequence was not available in GenBank) to make the analysis of the two datasets comparable. Fgb I7 sequences of *A. sylvaticus, M. domesticus* and *R. norvegicus* were available in GenBank (Accession nos. AY155330, EF605471 and AC105842) while *E. minor* and *N. rufus* Fgb I7 were directly sequenced by us from tissues samples (GenBank Accession nos. KF811250 and KF811251).

Phylogenetic relationships were estimated by Bayesian Inference. The analysis was conducted separately for cyt *b*, Fgb I7 and the combined dataset using Mr. Bayes v.3.1.2 [23]. For cyt *b*, the dataset was partitioned to correspond to the three codon positions. The appropriate evolution model for each partition of cyt *b* and, separately, for the Fgb I7 dataset was estimated using MrModeltest v.2.3 [24] following the AIC criteria [25]. The model of evolution that best describes the cyt *b* (for each partition) and Fgb I7 datasets was the GTR + I + Г (General Time Reversible [26]). Initially, the phylogenetic analysis was performed separately for the cyt *b* and Fgb I7 datasets, unlinking, on the cyt *b*, the model parameter, the base frequencies and the GTR for each partition under the same evolutionary model. Two independent Markov Chain Monte Carlo (MCMC) analyses were run with four chains and 1 million generations sampling the chains every 1000 generations. A burn-in of 10% of generated trees was applied. Furthermore, a partitioned Bayesian analysis was performed on the combined data matrix of the two markers. Four partitions were applied according to the structure and function of the markers (the three codon positions for cyt *b* and the Fgb I7). The analytical parameters of the Bayesian analysis applied to the separate markers were applied to the combined dataset.

For estimation of the divergence times among the species, a Relaxed Molecular Clock [27] was estimated on the combined dataset using the Bayesian method as implemented in BEAST v.1.4 [28]. Two fossil calibration points were taken from paleontological data [29], [30]: (1) the time of separation of *Steatomys* from *Dendromus* [30], dated between 8 and 11 mya; (2) the time of separation between the genera *Mus* and *Rattus* [31], dated between 10 and 12 mya. We performed the analysis using the uncorrelated relaxed clock with lognormal rate distribution. We employed the GTR + I + Г model on the four partitions of the dataset, with normally distributed priors for the parameters in models of molecular evolution, a Yule process tree model, and default values for all other settings. The MCMC was sampled 10,000 times every 100 cycles and the burn-in stage was set to 100,000 cycles.

### Cytogenetics and Zoo–FISH

One specimen for each of the following species was analysed cytogenetically: *D. melanotis* (voucher no. JW191, female from Bloemfontein), *D. mesomelas* (voucher no. DM8518, male from Dargle), *D. mystacalis* (voucher no. GD83, female from Mkuzi). Cell lines were established from rib and tail-tip clippings using conventional procedures; fibroblasts have been cryo-preserved in the Department of Botany and Zoology, Stellenbosch University. The diploid number (2n) and the number of autosomal chromosome arms (NFa) were determined for each species. Metaphases were G-banded [32] and C-banded [33] while Nucleolar Organizing Regions (NORs) were visualized by Ag-staining as described by Goodpasture and Bloom [34]. Each slide was previously counterstained with DAPI (Vector Laboratories) to identify the chromosomes bearing heterochromatin and NORs. For each staining, at least 10 metaphase spreads were analysed.

The G-banding of the short arm of chromosome X of all the species and the short arm of chromosome Y in *D. mesomelas* showed a band pattern of autosome 15 similar to that of *Mus musculus* [35], highlighting the possibility of autosome–sex chromosome translocation. Thus, Zoo-FISH analysis with *Mus musculus* (MMU) chromosome 15 paint biotin-labelled probe (Cambio Ltd.) was performed to confirm the presence of an autosomal segment on sex chromosomes. The probe of chromosome 15 of MMU was hybridized on two of the three species (*D. melanotis* and *D. mesomelas*) following the hybridization and detection procedure described in Rens [36]. Biotin-labelled probes were visualized using Cy3-Avidin (Amersham). Slides were mounted in Vectashield after pre-treatment with DAPI.

All the images were captured using the Genus Cytovision v.3.7 software (Applied Imaging Genetix, Queensway, UK).

## Results

### Molecular phylogenetic analysis

The total length of the alignment for cyt *b* is 1065 bp. We found 439 polymorphic sites, of which 89 are parsimoniously informative and 93 are at the third codon position. The base composition at the three codon positions follows the pattern described in Irwin et al. [17] for other mammals. In particular, at the second position, guanine and adenine have a low frequency (13% and 19% respectively) while thymine accounts for almost half of the nucleotides (42.1%). At the third codon position, guanine appears to be highly under-represented (2.1%) while adenine (39%) and cytosine (36.4%) are the most highly represented. The Fgb I7 alignment resulted in 621 aligned base pairs, of which 74 are variable sites and 48 are parsimoniously informative.

**Figure 2:**
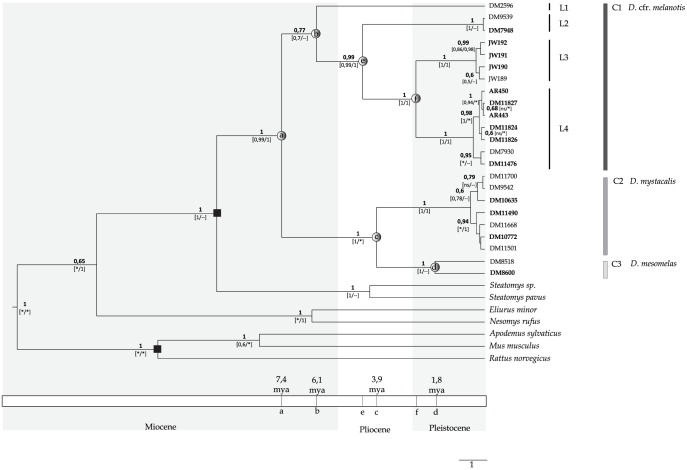
Phylogenetic tree and divergence times for South African *Dendromus*. Bayesian 50% majority rule consensus tree performed on the combined dataset (cyt *b* and Fib I7). The values at nodes indicate the posterior probability of the combined dataset (above the node) and the posterior probability of the corresponding node in the cyt *b* (on left, below the node) and Fib I7 (on right, below the node) trees. The asterisks (*) indicate incongruence at node; the double dash (–) indicate a node with a posterior probability less than 0,50. On the tree is also reported a time scale with the dating of the principal nodes (dots a – f) from relaxed molecular clock analysis.

The Bayesian 50% majority rule consensus tree performed on the combined dataset (cyt *b* + Fgb I7) resulted in most nodes being well-resolved with high posterior probability (PP) support (Fig. 2). *Dendromus* appears to be a monophyletic assemblage (PP = 1) and it is the sister taxon of *Steatomys*. Within the genus, three clades - C1 (PP = 0.77), C2 (PP = 1) and C3 (PP = 1) - are clearly recognizable. Clades C2 and C3 correspond to *D. mystacalis* and *D. mesomelas* respectively. Clade C1 contains all the individuals identified as *D. melanotis* and it is structured in four different well supported lineages (L1 - L4). Lineage L1 is represented by one specimen from Kamberg Nature Reserve in the KwaZulu - Natal Drakensberg area (locality 4); L2 (localities 6 and 9) and L4 (localities 7 and 5) are from continuous geographic regions. Finally, L3 includes all the specimens of *D. melanotis* from locality 2 (Table S1).

The cyt *b* consensus tree (not shown) shows the same topology as the one obtained from the combined dataset, except for two instances of terminal node conflict. The lineages L1-L4 are recognizable and well supported. The Fgb I7 consensus tree (not shown) confirmed the monophyly of the South African species. The tree highlights the presence of two clades and the single individual of *D. mesomelas* available for Fgb I7 is included within *D. mystacalis*. The distinction among lineages L2, L3 and L4 is also well supported for Fgb I7. We cannot confirm the same for L1 since we did not obtain the Fgb I7 sequence from the specimen representing that lineage.

The pairwise genetic divergence (Table 1) among the four lineages of C1 (lineages L1-L4) based on cyt *b* ranges between 10.6% and 18.3%. This divergence is considerable, comparable to the one between C2 and C3 (10.5%). The divergence within lineages L2, L3, L4 and within C2 and C3 is very low (0.4–0.6%). The two haplotypes constituting C1 (*D. mesomelas*) show high divergence (10.6%). For Fgb I7, the distances are reported in Table 1.

A relaxed molecular clock (Fig. 2) suggests that the first split in *Dendromus* occurred during the Late Miocene [node “a” 7.4 mya (95% CI: 5.4–9.1)] followed by the split between L1 and the ancestor of L2, L3 and L4 [node “b”, 6.1 mya (95% CI: 3.7–8.2)]. The split of *D. mesomelas* from *D. mystacalis* was more recent [node “c”, 3.9 mya (95% CI: 2.4–5.9)], comparable to the split between L2 and the ancestor of L3-L4 [node “e”, 4,4 mya (95% CI: 2,3 – 6,1)]. Other splits occurring within *D. mesomelas* as well as the split between L3 and L4 occurred in the late Pleistocene [node “d” 1.8 mya (95% CI: 0.4–3) and node “f”, 2.5 mya (95% CI: 1.3–3.7) respectively].

### Karyotypes

**Figure 3:**
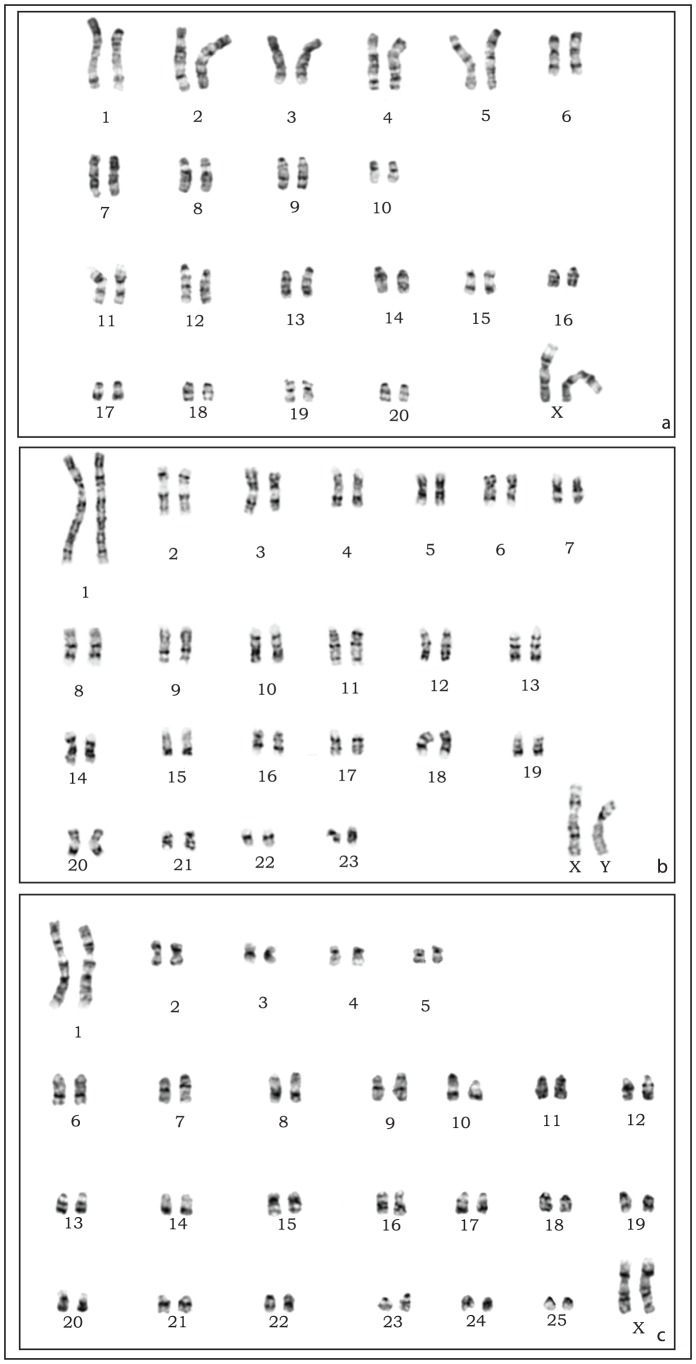
G Banding of South African *Dendromus*. G-banded karyotypes of (a) a female of *D. melanotis* (b) a male of *D. mesomelas* (c) a female of *D. mystacalis*.

The G-banded karyotypes are illustrated in Fig. 3. An example of sequential DAPI-C-banding and DAPI-Ag-NORs sequential banding is reported in Fig. S1.

- ***D.*** **cfr.** ***melanotis*** **, (lineage L3), 2n = 42.** The karyotype comprises 10 biarmed and 10 acrocentric pairs of chromosomes (Fig. 3a), FNA = 60. The biarmed can be divided into nine submetacentrics (nos. 1, 2, 3, 4, 5, 6, 7, 8 and 10) and one pair of metacentrics (no. 9). The X chromosome is a large submetacentric, comparable in size to autosomal pair 2. The C-banding pattern (Fig. S2a) shows that the short arms of the biarmed chromosomes (except 6, 7 and 9) are heterochromatic. The distal part of X_p_ is similarly heterochromatic. The Ag-nitrate staining shows the presence of NORs at telomeres on pairs 7 and 15, and in an interstitial position on the short arm on chromosomes 5 and 10 (Fig. S2d).
- ***D. mesomelas*** **, 2n = 48.** The karyotype comprises seven pairs of biarmed and 16 pairs of acrocentric autosomes (Fig. 3b), FNA = 60. The biarmed elements can be further classified as one pair of very large submetacentrics (no. 1) and six pairs of medium-sized meta-submetacentric chromosomes (nos. 2, 3, 4, 5, 6, 7). The X and Y are both large submetacentrics. C-banding () shows an interstitially located prominent C-positive band on X_q_ and Y_q_ to be entirely heterochromatic. On autosomes, heterochromatin is limited to paracentromeric bands on chromosomes 1, 2, 6, 14, 18. The Ag-staining highlights the presence of 8 NORs: one is interstitial on the short arm of chromosome 2, one is telomeric on chromosome 5, and both telomeric ends of chromosome 7 carry NORs (Fig. S2e).
- ***D. mystacalis*** **, 2n = 52.** The karyotype comprises five biarmed pairs and 20 acrocentric pairs of chromosomes (Fig. 3c), FNA = 60. The biarmed elements are represented by one pair of large submetacentrics (no. 1) and four pairs of small meta-submetacentrics (nos. 2, 3, 4 and 5). The X chromosome is a large submetacentric. The C-banding (Fig. S2c) reveals constitutive heterochromatin at the centromere of the autosomes. The X chromosome shows heterochromatin limited to the centromere. The Ag-nitrate staining reveals the presence of 12 NORs, in a centromeric position on chromosomes 8, 11, 13, 17, 23 and in a telomeric position on chromosome 9 (Fig. S2f).

### Sex chromosome comparison and Zoo–FISH

**Figure 4:**
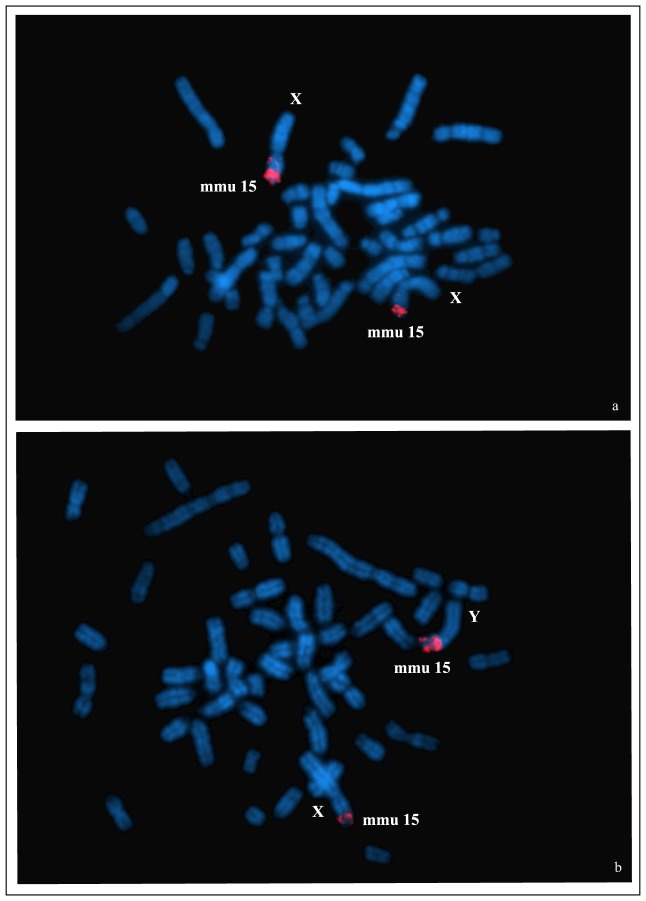
Autosome-sex chromosome translocation. Zoo FISH results of the *Mus musculus* chromosome 15 probe hybridization on the sex chromosomes in *D. melanotis* (a) and *D. mesomelas* (b).

The X chromosomes of the three specimens share the same G-banding patterns. The X_q_ shows a G-banding pattern typical of the euchromatic part of the X chromosome of many rodent species. Instead, X_p_ has a banding pattern comparable to chromosome 15 of the *Mus musculus* karyotype. The Y chromosome, studied only in *D. mesomelas*, presents a short arm that matches the *Mus musculus* autosome 15. The MMU15 probe hybridizes on the distal part (about 1/3) of the short arm of the X chromosomes of *D. melanotis* (Fig. 4a) and *D. mesomelas* (Fig. 4b) and, with the same proportion, on the distal part of the short arm of the Y chromosome in *D. mesomelas* (Fig. 4b).

## Discussion

### Nuclear and mitochondrial DNA diversity

The relationships among the studied species are well supported by both the combined and separate (cyt *b* and Fgb I7) datasets. The slight incongruence in terminal nodes between cyt *b* and Fgb I7 is probably due to the incomplete lineage sorting and to the presence of a single *D. mesomelas* individual for Fgb I7. The tree also shows a high support for three of the lineages constituting *D. melanotis* (L2, L3 and L4), with L1 constituted by one specimen. What is surprising is the high divergence among these lineages for cyt *b*, ranging from 10.6% to 18.3% and comparable to the value observed between *D. mesomelas* and *D. mystacalis*. These divergence values fall in the upper part of the distribution of cyt *b* divergences between sister species and clearly above the divergence found within rodent species [37]. Moreover, the Fgb I7 clusters, whose divergence is also considerable (2.4–4.1%), are reciprocally monophyletic with the mtDNA lineages. This raises the question of the taxonomic evaluation of lineages L1 to L4. According to Baker and Bradley [2], high intraspecific divergence (comparable to the one observed between species) and reciprocal monophyly between nuclear and mtDNA markers indicate the presence of “cryptic species”. Therefore, four putative cryptic species corresponding to L1 to L4 are present within *D. melanotis* [hereafter indicated as “*D.* cfr. *melanotis*”] and deserve additional studies. Moreover, it should be considered that these putative cryptic species might not be truly “cryptic”. In fact, Meester et al. [38] described three subspecies for *D. melanotis* in South Africa (*D. m. vulturnus*, *D. m. arenarius* and *D. m. melanotis*) as an indication of a certain morphological variability. Concerning the karyotypes, we suspect the presence of chromosomal variation. In fact, we studied only one specimen with 2n = 42. A different chromosomal formula, 2n = 52, is reported by Dippenaar et al. [15] from an unknown South African locality. Therefore, a comparative study of molecular, morphological and chromosomal variation of lineages L1-L4 within *D. melanotis* is required for a taxonomic revision of this species.

### Chromosomal evolution

Present and previous reports concur in indicating that the chromosomal evolution in *Dendromus* is characterized by an extended karyotype reorganization. This is highlighted both by the wide range of diploid (30<2n<52) and fundamental (42<FNa<96) numbers characterizing the different taxa (present data) [11], [13]–[15] and by the banding studies, limited only to four species (present data, [11]). The presented cytogenetic data are limited to 3 out the 6 taxa identified by molecular analysis (C2, C3 and L3 within *D.* cfr. *melanotis*) and additional studies are needed to investigate chromosomal evolution in detail. Nonetheless, we evidenced a general extended reorganization among the studied karyotypes. In fact, the comparison of G-banding patterns does not allow us to match most of the autosome/autosomal segments in the three species studied here, as well as in *D. lachaisei*. This may be partly due to the small size of many autosomes and/or to the different resolution of G-banding for the different species, but it also suggests an extensive reorganization of the karyotype by several complex rearrangements. In fact, in other African rodent genera, nearly complete identification of chromosomal homologies among species is possible with G-banding, notwithstanding the presence of chromosomal rearrangements - e.g., *Taterillus* [39]; *Arvicanthis* [40]; *Nannomys* [41]. However, it is not an exception in congeneric species that G-banding fails to reveal clear chromosomal homologies [42]. The only clear homology in the autosomal set is evident in pair 1 of *D. mesomelas* and *D. mystacalis*. This chromosome, very distinctive for its large size, appears identical in the two species under G-banding. A similar chromosome is also present in *D. lachaisei* but the centromeric index appears different, suggesting a chromosomal rearrangement [11]. It would be interesting to test if species carrying the distinctive chromosome pair 1 all belong to the same lineage and thus if it could be considered a chromosomal marker of phylogenetic significance. *D. mesomelas* and *D. mystacalis* also share the same fundamental number FNa = 60. Conversely, we were not able to pair any of the autosomes between *D.* cfr. *melanotis* (L3) and the other species. The distinction of the *D.* cfr. *melanotis* (L3) karyotype is also supported by a different pattern of heterochromatin distribution, with heterochromatic arms on six pairs of chromosomes. This pattern is lacking in the *D. mesomelas* and *D. mystacalis* specimens studied here as well as in *D. lachaisei* [11].

It is likely that the differences among the species in number and position of NORs could be also the result of the chromosomal reorganization occurring during the species divergence. Nonetheless, it is impossible to exclude that the Ag-staining revealed false NORs. Indeed, it has been shown that the Ag-staining technique is not always a good means to reveal the correct position of NORs in all mammalian groups [43]. This karyotype variability among main clades leads to the hypothesis of a presence of karyotypic variability even among the lineages of *D*. cfr. *melanotis*. The definition of the karyotype of *D*. cfr. *melanotis*, as its phylogenetic status, deserves strengthening by providing the karyotype of at least one animal for each lineage highlighted in this work.

The observed autosome repatterning contrasts with the conservatism of the shared autosome-sex chromosome translocation characterizing all the species studied so far. In fact, the FISH with the chromosome 15 *Mus* probe confirmed the presence of autosomal segments on the X chromosome in *D.* cfr. *melanotis* (L3) and on both X and Y of *D. mesomelas*. The X chromosome of *D. lachaisei* from Guinea has the same G-banding pattern, suggesting that this species also has the same rearrangement [11]. The addition of an autosome onto the X (not associated with a Y-autosome translocation), lead to a XY1Y2 condition where Y1 corresponds to the true Y and the Y2 to the non-translocated copy of the autosomal segment. Such a system is not so uncommon in mammals (e.g., [41], [44] and reference therein). Conversely, the fixation of non-reciprocal Y-autosome translocation (not associated with an X-autosome translocation) is relatively rare (e.g., Deuve et al. [45]). Both of these systems may represent the first step toward the acquiring of the same autosomal translocation on both sex chromosomes by recombination [46] as observed in *Dendromus*. Autosome-sex chromosome translocations are supposed to occur with low frequency in mammals because of their highly deleterious effects on gene expression and gametogenesis [44]. Moreover, the possibility of retromutation is very low when the cytological mechanisms preventing the deleterious effects are setting up. Thus, when autosome-sex chromosome translocations are present in mammals, they are usually retained by all species within a lineage (references in [44]). In fact, sex-autosome translocations involving both the X and the Y chromosomes, as here observed in *Dendromus*, are documented in other mammalian species ([44], [47] and references therein). Our estimate from the molecular clock indicates that this mutation is rather ancient since it occurred at least about 7.4 mya in the Miocene. Additional species should be studied to confirm if X-autosome translocation characterizes the entire genus and if it could be even older.

### Biogeography/times of evolution

The Bayesian relaxed molecular clock suggests that dichotomic events in *Dendromus* took place in different phases, with two splits occurring at the end of the Miocene and the others during the Plio-Pleistocene. This concurs with a study on nine rodent genera which concluded that the majority of dichotomic events occurred during the last 5.3 Myr and only 17% preceded the Pliocene boundary [7]. It is believed that species evolution related to these events was not only linked to climatic oscillation but particularly relevant seems the interaction of these with the species life history and topographic barriers in the region [7]. *D. melanotis* lives in drier areas with short grassland whereas *D. mystacalis* and *D. mesomelas*, in sympatry in the same biome, prefer wet zones with long grass and forests. A similar pattern is observed in *Otomys* [48] and *Rhabdomys* [49] which currently include mesic clades located in western South Africa and xeric groups in the east of the country. These clades are probably the result of initial isolation in refugia, followed by differential adaptation to the predominantly surrounding mesic or dry habitat. Subsequently, the diversification process was reinforced by precipitation that shifted continuously during glacial cycles, leading to continuous expansion/regression of the vegetation types [50]. This could have triggered the split of the ancestor of *D. mystacalis* and *D. mesomelas* which now show a spatial and ecological separation that reflects parameters such as grass height and humidity [51]. A detailed distribution mapping of lineages L1 to L4 within *D. melanotis* is required to assess eventual ecological preferences in order to infer the additional speciation events among the *D.* cfr. *melanotis* lineages.

## Supporting Information

Figure S1 Examples of sequential DAPI-C-banding and DAPI-Ag-NORs sequential banding. From left to right: DAPI counterstained metaphases (a) are converted in inverted DAPI (b), by an image editing software, to visualize the banding pattern and identified the chromosomes. Afterwards, the same metaphase is C- banded or Ag-stained (c).
(PDF)

Figure S2 C-banded and Ag-stained karyotypes and mitotic metaphases of *D. melanotis* (a and d, respectively) *D.mesomelas* (b and e, respectively) and *D. mystacalis* (c and f, respectively).
(DOC)

Table S1 Table of the samples used in that study. For each individuals (voucher number), species, diploid numbers (2n), details on collecting site (Province, Area, Locality), locality code (see the map, figure 1), latitude and longitude for each locality, the cyt b and Fib I7 GenBank accession numbers are reported.
(DOC)
