# Supplementary material for: Cryptic Speciation and Chromosomal Repatterning in the South African Climbing Mice Dendromus (Rodentia, Nesomyidae)
Source: PLoS One. 2014 Feb 13;9(2):e88799. doi: 10.1371/journal.pone.0088799 (PMC3923822; doi:10.1371/journal.pone.0088799)
Supplement: Figure S1 — Examples of sequential DAPI-C-banding and DAPI-Ag-NORs sequential banding. From left to right: DAPI counterstained metaphases (a) are converted in inverted DAPI (b), by an image editing software, to visualize the banding pattern and identified the chromosomes. Afterwards, the same metaphase is C- banded or Ag-stained (c). (PDF) [file pone.0088799.s001.pdf]

Supporting Information for

Cryptic speciation and chromosomal repatterning in the South African Climbing mice *Dendromus* (Rodentia, Nesomyidae)

Emanuela Solano[1]*, Peter J Taylor [2,3], Anita Rautenbach[3], Anne Ropiquet [4], Riccardo Castiglia[1]

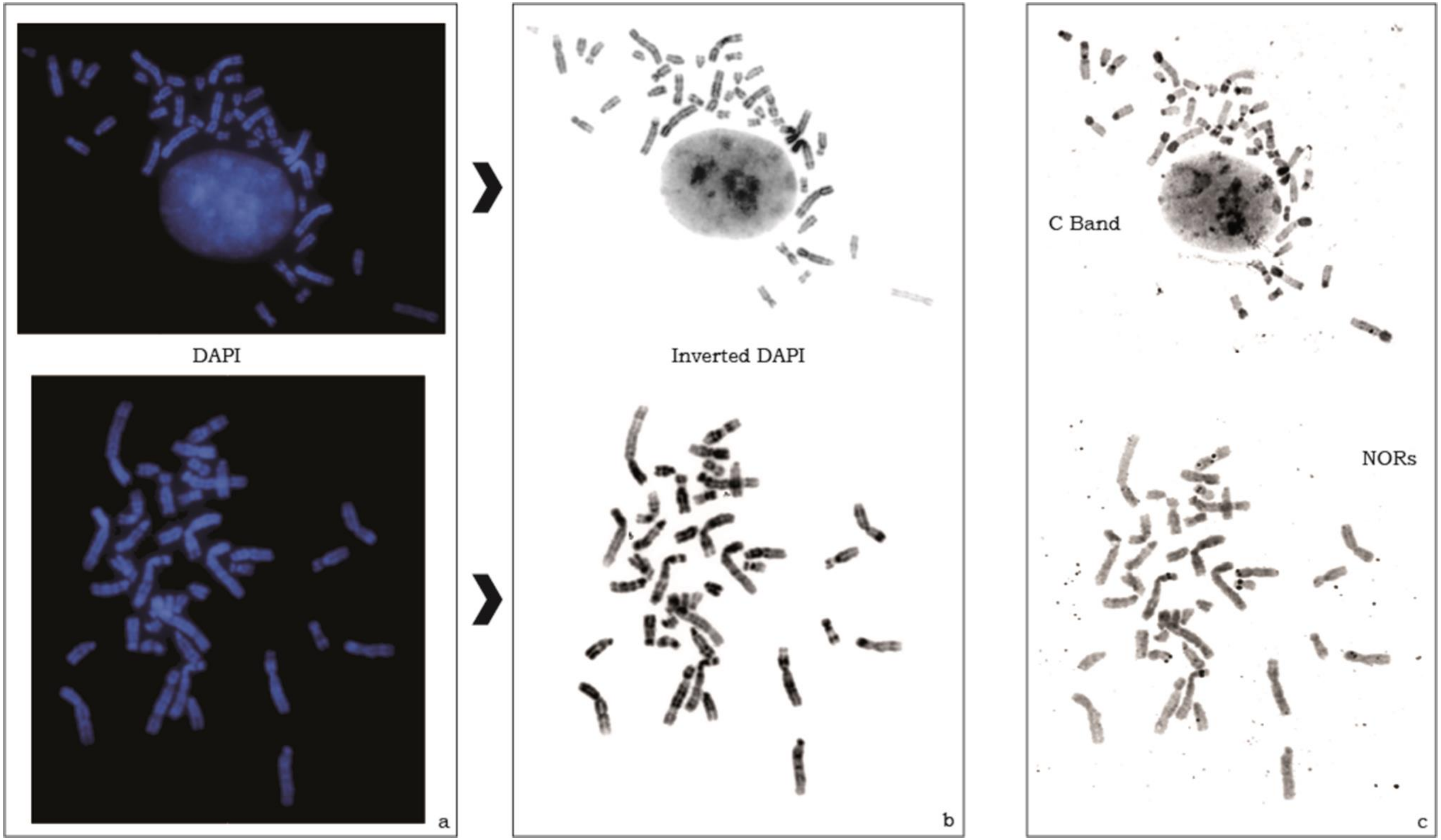
DAPI
a
Inverted DAPI
b
C Band
NORs
c
