# Supplementary material for: Cryptic Speciation and Chromosomal Repatterning in the South African Climbing Mice Dendromus (Rodentia, Nesomyidae)
Source: PLoS One. 2014 Feb 13;9(2):e88799. doi: 10.1371/journal.pone.0088799 (PMC3923822; doi:10.1371/journal.pone.0088799)
Supplement: Figure S2 — C-banded and Ag-stained karyotypes and mitotic metaphases of D. melanotis (a and d, respectively) D.mesomelas (b and e, respectively) and D. mystacalis (c and f, respectively). (DOC) [file pone.0088799.s002.doc]

Supporting Information for

**Cryptic speciation and chromosomal repatterning in the South African Climbing mice *Dendromus* (Rodentia, Nesomyidae)**

| Emanuela Solano^1^*, Peter J Taylor ^2,3^, Anita Rautenbach^3^, Anne Ropiquet ^4^, Riccardo Castiglia^1^ |
| --- |
| 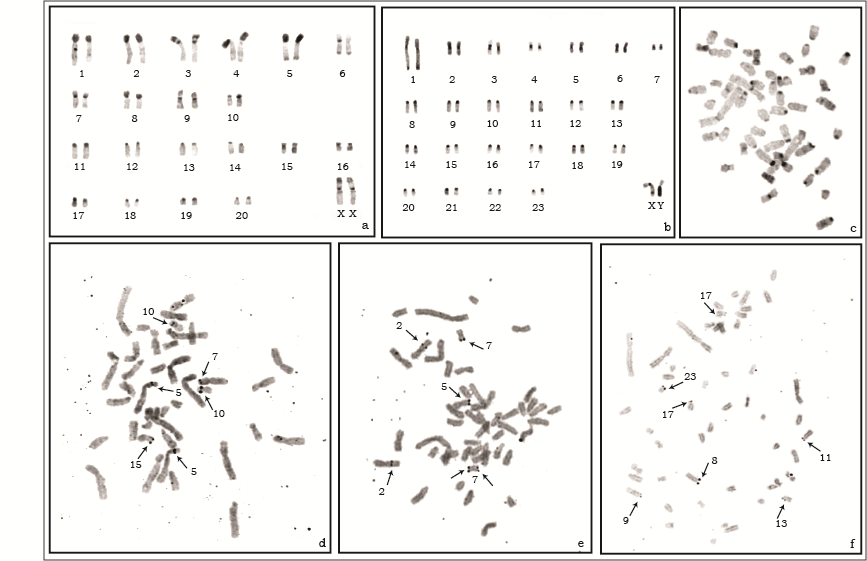 |
