# Supplementary material for: Cryptic Speciation and Chromosomal Repatterning in the South African Climbing Mice Dendromus (Rodentia, Nesomyidae)
Source: PLoS One. 2014 Feb 13;9(2):e88799. doi: 10.1371/journal.pone.0088799 (PMC3923822; doi:10.1371/journal.pone.0088799)
Supplement: Table S1 — Table of the samples used in that study. For each individuals (voucher number), species, diploid numbers (2n), details on collecting site (Province, Area, Locality), locality code (see the map, figure 1), latitude and longitude for each locality, the cyt b and Fib I7 GenBank accession numbers are reported. (DOC) [file pone.0088799.s003.doc]

Supporting Information for **Cryptic speciation and chromosomal repatterning in the South African Climbing mice *Dendromus* (Rodentia, Nesomyidae)**

Emanuela Solano^1^*, Peter J Taylor ^2,3^, Anita Rautenbach^3^, Anne Ropiquet ^4^, Riccardo Castiglia^1^

**Table S1** Table of the samples used in that study. For each individuals (voucher number), species, diploid numbers (2n), details on collecting site (Province, Area, Locality), locality code (see the map, figure 1), latitude and longitude for each locality, the cyt b and Fib I7 GenBank accession numbers are reported.

| **Species** | **Voucher number** | **2n** | **Province** |  | **Locality** | **Locality code** | **latitude** | **longitude** | **Fib I7** | **cyt *b*** |
| --- | --- | --- | --- | --- | --- | --- | --- | --- | --- | --- |
| *D. melanotis* | JW189 |  | Freestate | Bloemfontein | Bloemfontein | (2) | 29° 07'S | 26° 12'E |  | KF811226 |
| *D. melanotis* | JW190 |  | Freestate | Bloemfontein | Bloemfontein | (2) |  |  | KF811242 | KF811227 |
| *D. melanotis* | JW191 | 42 | Freestate | Bloemfontein | Bloemfontein | (2) |  |  | KF811243 | KF811228 |
| *D. melanotis* | JW192 |  | Freestate | Bloemfontein | Bloemfontein | (2) |  |  | KF811244 | KF811229 |
| *D. melanotis* | DM2596 |  | KwaZulu-Natal | Drakensberg | Kamberg | (4) | 29° 21'S | 29° 45'E |  | KF811213 |
| *D. melanotis* | DM7948 |  | KwaZulu-Natal | Drakensberg | Manzengwenya | (9) | 27° 13'S | 32° 46'E | KF811249 | KF811235 |
| *D. melanotis* | DM9539 |  | KwaZulu-Natal | Mkhuze | Mkhuze | (6) | 27° 34'S | 32° 14'E |  | KF811214 |
| *D. melanotis* | DM7930 |  | KwaZulu-Natal | St. Lucia | Mfabeni Wilderness Area | (5) | 28° 4'S | 32° 31'E |  | KF811220 |
| *D. melanotis* | AR443 |  | KwaZulu-Natal | Phinda | Phinda GR | (7) | 27° 47'S | 32° 18'E | KF811240 | KF811221 |
|  |  |  |  |  |  |  |  |  |  |  |
| *D. mesomelas* | DM 8518 | 48 | KwaZulu-Natal | Mpumalanga | Dargle | (3) | 29° 28'S | 30° 03'E |  | KF811215 |
| *D. mesomelas* | DM 8600 |  | Western Cape | Cedarberg | Uitkyk Pass | (1) | 32° 24'S | 19° 05'E | KF811247 | KF811233 |
|  |  |  |  |  |  |  |  |  |  |  |
| *D. mystacalis* | DM 9542 |  | KwaZulu-Natal | Mkhuze | Mkhuze | (6) |  |  |  | KF811219 |
| *D. mystacalis* | DM11501 | 52 | KwaZulu-Natal | Mkhuze | Mkhuze | (6) |  |  |  | KF811218 |
| *D. mystacalis* | DM11490 |  | KwaZulu-Natal | Phinda | Phinda GR | (7) |  |  | KF811248 | KF811234 |
| *D. mystacalis* | DM10772 |  | KwaZulu-Natal | Mkhuze | Mkhuze | (6) |  |  | KF811246 | KF811232 |
| *D. mystacalis* | DM10635 |  | KwaZulu-Natal | Mkhuze | Mkhuze | (6) |  |  | KF811245 | KF811231 |
| *D. mystacalis* | DM11700 |  | KwaZulu-Natal | Kube Yini | Kube Yini GR | (8) | 27° 48'S | 32° 14'E |  | KF811216 |
| *D. mystacalis* | DM11668 |  | KwaZulu-Natal | Kube Yini | Kube Yini GR | (8) |  |  |  | KF811217 |
|  |  |  |  |  |  |  |  |  |  |  |
| *Dendromus sp.* | DM11476 |  | KwaZulu-Natal | Phinda | Phinda GR | (7) |  |  | KF811239 | KF811230 |
| *Dendromus sp.* | AR450 |  | KwaZulu-Natal | Phinda | Phinda GR | (7) |  |  | KF811241 | KF811224 |
| *Dendromus sp.* | DM11824 |  | KwaZulu-Natal | Phinda | Phinda GR | (7) |  |  | KF811237 | KF811223 |
| *Dendromus sp.* | DM11826 |  | KwaZulu-Natal | Phinda | Phinda GR | (7) |  |  | KF811236 | KF811222 |
| *Dendromus sp.* | DM11827 |  | KwaZulu-Natal | Phinda | Phinda GR | (7) |  |  | KF811238 | KF811225 |
|  |  |  |  |  |  |  |  |  |  |  |
